# Associations of accelerometer-measured physical activity, sedentary behaviour, and sleep with next-day cognitive performance in older adults: a micro-longitudinal study

**DOI:** 10.1186/s12966-024-01683-7

**Published:** 2024-12-10

**Authors:** Mikaela Bloomberg, Laura Brocklebank, Aiden Doherty, Mark Hamer, Andrew Steptoe

**Affiliations:** 1 Department of Epidemiology and Public Health, University College London, London, UK; 2 Nuffield Department of Population Health, University of Oxford, Oxford, UK; 3 Division of Surgery and Interventional Science, University College London, London, UK; 4 Department of Behavioural Science and Health, University College London, London, UK

**Keywords:** Cognitive function, Cognitive ageing, Sleep, Physical activity, Sedentary behaviour

## Abstract

**Background:**

Previous studies suggest short-term cognitive benefits of physical activity occurring minutes to hours after exercise. Whether these benefits persist the following day and the role of sleep is unclear. We examined associations of accelerometer-assessed physical activity, sedentary behaviour, and sleep with next-day cognitive performance in older adults.

**Methods:**

British adults aged 50-83 years (*N* = 76) without evidence of cognitive impairment or dementia wore accelerometers for eight days, and took daily cognitive tests of attention, memory, psychomotor speed, executive function, and processing speed. Physical behaviour (time spent in moderate-to-vigorous physical activity [MVPA], light physical activity [LPA], and sedentary behaviour [SB]) and sleep characteristics (overnight sleep duration, time spent in rapid eye movement [REM] sleep and slow wave sleep [SWS]) were extracted from accelerometers, with sleep stages derived using a novel polysomnography-validated machine learning algorithm. We used linear mixed models to examine associations of physical activity and sleep with next-day cognitive performance, after accounting for habitual physical activity and sleep patterns during the study period and other temporal and contextual factors.

**Results:**

An additional 30 min of MVPA on the previous day was associated with episodic memory scores 0.15 standard deviations (SD; 95% confidence interval = 0.01 to 0.29; *p* = 0.03) higher and working memory scores 0.16 SD (0.03 to 0.28; *p* = 0.01) higher. Each 30-min increase in SB was associated with working memory scores 0.05 SD (0.00 to 0.09) lower (*p* = 0.03); adjustment for sleep characteristics on the previous night did not substantively change these results. Independent of MVPA on the previous day, sleep duration ≥ 6 h (compared with < 6 h) on the previous night was associated with episodic memory scores 0.60 SD (0.16 to 1.03) higher (*p* = 0.008) and psychomotor speed 0.34 SD (0.04 to 0.65) faster (*p* = 0.03). Each 30-min increase in REM sleep on the previous night was associated with 0.13 SD (0.00 to 0.25) higher attention scores (*p* = 0.04); a 30-min increase in SWS was associated with 0.17 SD (0.05 to 0.29) higher episodic memory scores (*p* = 0.008).

**Conclusions:**

Memory benefits of MVPA may persist for 24 h; longer sleep duration, particularly more time spent in SWS, could independently contribute to these benefits.

**Supplementary Information:**

The online version contains supplementary material available at 10.1186/s12966-024-01683-7.

## Background

Ageing-related decline in cognitive function is an important predictor of loss of autonomy and reduction in wellbeing for older adults [1]. Identifying modifiable behavioural factors that contribute to the maintenance of daily cognitive function for older adults is therefore of utmost importance to maintain quality of life and active social participation and to mitigate cognitive symptoms for adults living with cognitive impairment and dementia. Physical activity is one such behavioural factor identified as protective for cognitive function [2, 3], where individuals who habitually participate in more physical activity may have a reduced rate of cognitive decline with ageing and reduced dementia risk [2, 4]. Exercise is also linked with short-term improvements in cognitive performance [5]. Accordingly, sedentary behaviour at the expense of physical activity is linked with worse cognitive performance [6]. Laboratory-based studies suggest the largest exercise-induced improvements in cognitive performance occur in the minutes to hours immediately following exercise [7, 8], and are attributable to increased blood flow to the brain and stimulation of neurotransmitters [5].

Accumulating acute cognitive benefits of physical activity could contribute to mitigation of cognitive decline. However, whether acute cognitive benefits of physical activity persist—even to the following day—is not yet known. Several studies use ecological momentary assessments to examine cognitive performance and accelerometery-assessed physical activity to suggest links between physical activity and same-day improvements in cognitive performance [9–12]. One study found associations between physical activity and working memory performance on the following day in pre-adolescents, but did not examine adults [10]. These studies do not consider the role of sleep. Physical activity may influence sleep duration [13], which is itself independently associated with cognitive performance, cognitive decline, and risk of dementia [14–16]. Several studies also find sleep stage specific associations with cognitive performance, where rapid eye movement (REM) sleep deprivation is associated with impairments to nondeclarative memory, while non-REM stage III (also referred to as slow wave sleep or SWS) sleep deprivation is associated with impairments to declarative memory [17–21]. Physical activity has been found to promote SWS and delay onset of REM sleep [22–25].

To elucidate next-day cognitive benefits of physical activity and sleep, we used eight days of wrist-worn accelerometer data from 76 British adults aged 50-83 years who took online cognitive tests on each day of accelerometer wear. We examined associations of physical activity (time spent in moderate-to-vigorous physical activity [MVPA] and light physical activity [LPA]), sedentary behaviour (SB), and sleep (overnight sleep duration, time spent in REM sleep and SWS) with next-day cognitive performance in five cognitive domains (attention, memory [episodic memory and working memory], psychomotor speed, executive function, and processing speed). We hypothesised that more physical activity, less sedentary behaviour, and longer sleep would be associated with better cognitive performance on the following day.

## Methods

### Data sources

A convenience sample of 85 adults able to walk around with or without an aid, aged 50 + years, living in the UK, fluent in English, and with internet access were recruited via email using university contacts or word of mouth as part of a micro-longitudinal free-living validation study aiming to establish equivalency between two brands of wrist-worn accelerometer (Axivity AX3 and the newly developed Matrix 003). A micro-longitudinal study refers to longitudinal studies undertaken over hours or days rather than months or years (see for example, Zhang et al. 2021, Dautovich et al. 2013 [26, 27]). For the present analysis, data from the validated Axivity AX3 accelerometer [28, 29] were used. A secondary aim of the micro-longitudinal study was to examine day-to-day associations of movement behaviours (physical activity, sedentary behaviour, and sleep) with cognitive health and mental wellbeing; as such, participants were also asked to take daily cognitive tests. The study was approved by the University College London Research Ethics Committee (Reference: 20243/001). Participants provided written informed consent. Data were collected between October 2021 and November 2022.

### Accelerometer data collection

Participants were asked to start wearing the accelerometer immediately after receiving it in the post and wear it on their dominant wrist 24 h per day for eight consecutive days. Participants were informed that they could wear the waterproof accelerometer when bathing or swimming but not in extremely high temperature or pressure environments (e.g., in a sauna or when diving) and were asked to carry on with their normal activities. Participants were not given feedback on their activity levels until after the accelerometer was returned.

The Axivity AX3 (Axivity Ltd, Newcastle, UK) is a validated wrist-worn triaxial accelerometer that has been used to measure 24-h movement behaviours (physical activity, sedentary behaviour, and sleep) in the UK Biobank [29] and the China Kadoorie Biobank [28]. The Axivity was set to start recording at 10am two working days after postal dispatch and capture triaxial acceleration data at 100 Hz with a dynamic range of ± 8 *g*.

Physical activity and sedentary behaviour patterns were derived from raw acceleration data using the open-source Biobank Accelerometer Analysis Tool (https://github.com/OxWearables/biobankAccelerometerAnalysis, v7.0.1), which was developed and validated by the Oxford Wearables Group (Big Data Institute, University of Oxford). Similar procedures as those used in the UK Biobank were used to process the raw acceleration data [29]. Participants were excluded if the data could not be parsed, the device could not be calibrated, more than 1% of readings fell outside the device’s dynamic range of ± 8 *g* before or after calibration, or the average acceleration was implausibly high (> 100 m*g*). The Biobank Accelerometer Analysis Tool was used to produce the percentage of the 24-h day spent in MVPA, LPA, and SB, which was then converted into hours per day. For days with wear time less than 22 h (7 of 291 days for the present analysis with a median wear time of 20.0 h, interquartile range = 10.0–21.6 for days with < 22 h), we imputed time spent in MVPA, LPA, and SB using the weekday or weekend mean proportion for that individual, with non-wear time defined as unbroken episodes of at least 60 min during which the standard deviation (SD) of each axis of acceleration was less than 13 m*g* [29].

Sleep quality characteristics were derived using an open-source sleep staging algorithm (SleepNet: https://github.com/OxWearables/asleep, v0.4.12, which was also developed and validated using polysomnography by the Oxford Wearables Group [30]. SleepNet classifies each 30-s window of acceleration data into sleep stages (wake, REM, non-REM stages I or II, or SWS). For SleepNet, participants were excluded if the data could not be parsed, the device could not be calibrated, more than 1% of readings fell outside ± 3 *g* before or after calibration, or the average acceleration was implausibly high (> 200 m*g*). SleepNet excludes days if the participant wore the accelerometer for less than 22 h, based on evidence suggesting at least 22 h of wear time was required for stable weekly sleep parameter estimates [31], with non-wear time defined as unbroken episodes of at least 90 min during which the SD of each axis of acceleration was less than 13 m*g*.

SleepNet was used to determine sleep parameters for the longest sleep window over a noon-to-noon interval, with up to 60 min of sleep discontinuity allowed. In the present study, the sleep parameters we examined were overall sleep duration and minutes spent in REM sleep and SWS; we focussed on these parameters based on the large body of evidence suggesting their association with cognitive function [14, 16–21]. Overall sleep duration was categorised into short (2- < 6 h) or optimal (≥ 6 h) based on evidence that 6–8 h of sleep per night is optimal for cognitive performance [14]. As sleep durations longer than 8.7 h accounted for just 5% of observations, we did not include a ‘long sleep duration’ category.

### Cognitive function

**Fig. 1:**
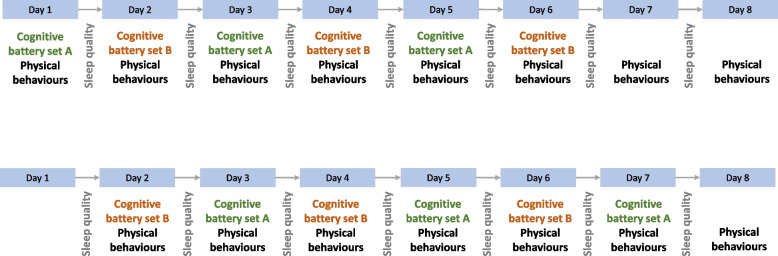
Two example timelines of data collection. Cognitive battery set A: cancellation test and picture recall. Cognitive battery set B: Corsi block-tapping test, simple reaction time, Trail Making Test B. Physical behaviours include time spent in moderate-vigorous physical activity, light physical activity, and sedentary behaviour. Sleep quality characteristics include overall sleep duration and time spent in rapid eye movement sleep and slow wave sleep

There were two sets of cognitive tests (set A and set B) self-administered using an online platform (Neuropsychology Online [NeurOn]: https://neuropsychology.online/). Participants took a set of cognitive tests every day, alternating between set A and set B in a random order to reduce learning effects (Fig. 1). Participants were asked to complete the cognitive tests at the same time each day, using the same type of device (computer, tablet, or smartphone), and using the same input method (keyboard and mouse or touchscreen). Participants were not given feedback on their cognitive scores until after the accelerometer was returned.

Cognitive domains assessed were those that have been previously demonstrated to be associated with physical activity and sleep quality [8, 17, 19, 32–34], including attention (the cancellation test [35]), memory (episodic memory using picture recall and working memory using the Corsi block-tapping test [36]), psychomotor speed (simple reaction time [37]), executive function (Trail Making Test B [38]), and processing speed (total time to complete Trail Making Test B [38]).

Cognitive battery set A included the cancellation test and picture recall. Set B included the Corsi block-tapping test, simple reaction time, and Trail Making Test B. In the cancellation test, participants were asked to scan through an array of different symbols and select all occurrences of one particular symbol. Participants were scored on the number of symbols correctly selected of the total number of symbols they were supposed to select, which was converted to a percentage. In the picture recall task, participants were shown a series of pictures and then after a short delay asked whether they had been shown that picture previously. The picture recall task was scored as a percentage corresponding to the number of correctly identified pictures minus the number of pictures incorrectly identified out of the total number of pictures shown. In the Corsi block-tapping task, squares were highlighted with an increasing sequence ranging from two to nine. Participants were asked to correctly recall the order in which the squares were highlighted and were scored on the highest sequence they were able to repeat correctly on both attempts. The simple reaction time task required participants to react to a stimulus as fast as possible, with time measured in milliseconds. Finally, in trail making test B, participants were asked to click alternating numbers and letters in ascending order as fast as possible. Participants were scored on accuracy (as a percentage) and total time (in seconds). To facilitate interpretation of the results, cognitive scores were standardised using the mean and SD of the analytic sample.

### Other covariates

At the start of the wear period, participants were asked to report their gender (man or woman), age in years, highest educational qualification (no qualifications, O-level, A-level, further education below university degree, university degree, or postgraduate degree), self-rated health (excellent, very good, good, fair, or poor), number of mobility limitations (0–10; Table S1), employment status (employed or not), and height and weight which was used to produce body mass index (BMI; kg/m^2^). Depressive symptoms were measured at the start of the wear period using the 10-item Center for Epidemiological Studies-Depression (CES-D) Scale with scores ranging from 0-20 and a higher score indicating more depressive symptoms [39]. Other covariates were recorded daily and included the time of day cognitive tests were taken (categorised into morning [06:00-11:59], afternoon [12:00-16:59], or evening [17:00-22:00]), round of cognitive testing (to account for learning effects), and whether it was a weekday or not. For two participants missing BMI, we singly-imputed BMI using linear regression models with gender, age, education, self-rated health, mobility limitations, CES-D score, and mean physical activity and sedentary behaviour during the follow-up period as predictors. No other covariates were missing.

### Statistical analysis

We used linear mixed models to examine associations of physical activity, sedentary behaviour, and sleep with cognitive scores, with separate models used for each cognitive domain and for each movement behaviour. Linear mixed models use all available observations and account for data missing-at-random [40]. In the present analysis, models included a random intercept at the individual level to account for intraindividual correlation of repeated measurements; each participant contributed up to two measurements per cognitive test.

To estimate the total effect of the previous day’s movement behaviours on cognitive performance, the minimally sufficient adjustment set for each set of models (physical activity, sedentary behaviour, or sleep models) was identified based on directed acyclic graphs (DAGs; Figures S1-S2). Based on these DAGs, for cognitive outcome $y$ at time $t$, ${y}_{t}$, physical activity and sedentary behaviour models were adjusted for sleep parameters at $t-2$ and cognitive performance at $t-2$. Sleep models (overall sleep duration, REM sleep, SWS) were adjusted for physical activity and sedentary behaviour at time $t-1$ and cognitive performance at $t-2$. All models were also adjusted for physical activity at time $t$ to account for participants engaging in physical activity before taking the cognitive test at time $t$, as it is already established that physical activity affects cognitive performance in the minutes to hours following a bout of physical activity [5, 7, 8].

It is also well-established that individuals who habitually participate in more physical activity [2, 3, 5, 7, 8] or who generally have better sleep quality [13–15] have better cognitive performance. We therefore focussed on daily fluctuations in movement behaviours by adjusting for habitual physical activity, sedentary behaviour, and sleep habits during the wear period. In addition to the physical activity, sedentary behaviour, sleep, and cognitive covariates identified using DAGs, all models were adjusted for age, gender, education, mobility limitations, self-rated health, depressive symptoms, employment status, BMI, time-of-day of cognitive test taking, round of cognitive testing, weekend or weekday, habitual physical activity and sedentary behaviour (mean time spent in MVPA, LPA, and SB during the monitoring period for that individual), and habitual sleep (mean overall sleep duration, REM sleep, and SWS during the monitoring period for that individual). Gender, employment status, time-of-day of test taking, and weekend or weekday were included in models as categorical variables; all other covariates were included as continuous variables. Details of variable coding are available in the Supplemental materials (Table S2). In line with recommended procedure [41], as we focussed on cognitive domains that have established associations with physical activity and sleep and did not perform post-hoc re-analyses, we did not account for multiple comparisons.

### Additional analyses

To estimate the association between the previous day’s MVPA and cognitive performance independent of the previous night’s sleep, we additionally adjusted physical activity and sedentary behaviour models for overall sleep duration at $t-1$, and time spent in REM sleep and SWS at $t-1$. We do not include sleep models unadjusted for physical behaviours because physical behaviours confound the association between sleep and next-day cognitive performance, whereas sleep is along the causal pathway from physical behaviours to next-day cognitive performance.

## Results

### Sample characteristics

Of 85 participants, two (2.3%) only had one set of cognitive tests, and seven (8.2%) were missing movement behaviour data due to failing quality checks and were excluded, leading to 76 participants included in the analysis. Characteristics of the analytic sample are presented in Table 1. Of 76 participants, 46 (60.5%) were women. The mean age of participants was 64.6 years (SD = 10.0). Participants were highly educated (64 [84.2%] with educational qualifications above A-level), with none reporting poor health and 71 (93.4%) reporting good, very good, or excellent health. The median number of mobility limitations was 0 (interquartile range [IQR] = 0-0) and the median depressive symptom score was 5 (IQR = 2-8). The 76 participants contributed 291 observations, during which the majority took cognitive tests in the afternoon (176 [60.5%) of observations) or morning (101 [34.7%]). The mean daily time spent in MVPA, LPA, and SB was 0.9 h (SD = 0.9), 5.1 h (SD = 2.1), and 9.2 h (SD = 2.3) respectively. The mean overnight sleep duration was 7.0 h (SD = 1.2), and the mean time spent in REM sleep and SWS was 1.5 h (SD = 0.8) and 1.9 h (SD = 0.7) respectively. Mean raw scores for each cognitive domain are reported in Table 1.

**Table 1:** Characteristics of the analytic sample (*N* = 76)

*Gender*	
Male	46 (60.5)
Female	30 (39.5)
*Mean age (SD)*	64.6 (10.0)
*Educational qualifications*	
A level or below	12 (15.8)
Above A level to university degree	47 (61.8)
Postgraduate degree	17 (22.4)
*Self-rated health*	
Excellent	14 (18.4)
Very good	37 (48.7)
Good	20 (26.3)
Fair	5 (6.6)
Poor	0 (0.0)
*Median number mobility limitations (IQR)*	0 (0-0)
*Median depressive score (IQR)*	5 (2-8)
*Physical activity and sedentary behaviour*	
Mean hours in MVPA (SD)	0.9 (0.9)
Mean hours in LPA (SD)	5.1 (2.1)
Mean hours in SB (SD)	9.2 (2.3)
*Overnight sleep*	
Mean sleep duration in hours (SD)	7.0 (1.2)
Mean hours in REM sleep (SD)	1.5 (0.8)
Mean hours in SWS (SD)	1.9 (0.7)
*Mean cognitive performance (SD)*	
Attention (%)	96.0 (6.6)
Episodic memory (%)	85.7 (14.0)
Working memory (highest sequence)	4.7 (1.2)
Psychomotor speed (milliseconds)	330 (56.2)
Executive function (%)	88.8 (20.5)
Processing Speed (seconds)	53.6 (29.4)

Data shown are N (%) unless otherwise indicated

*Abbreviations*: *SD* standard deviation, *IQR* interquartile range, *MVPA* moderate-vigorous physical activity, *LPA* light physical activity, *SB* sedentary behaviour, *REM* rapid eye movement, *SWS* slow wave sleep

### Physical activity and sedentary behaviour

Before adjustment for the previous night’s sleep quality, time spent in MVPA on the previous day was associated with cognitive scores for episodic and working memory (Table 2). Each additional 30 min of MVPA on the previous day was associated with a 0.15 SD (95% confidence interval [CI] = 0.01 to 0.29) increase in score for episodic memory (*p* = 0.03). Each additional 30 min of MVPA on the previous day was also associated with a 0.16 SD (0.03 to 0.28) increase in working memory score (*p* = 0.01). Each 30-min increase in MVPA on the previous day corresponded to a 0.09 SD (0.00 to 0.18) improvement in performance for psychomotor speed; however, this improvement did not reach statistical significance (*p* = 0.06). Additional MVPA on the previous day was not associated with cognitive scores for attention (per 30-min increase in MVPA, change in cognitive score = 0.02 SD, 95% CI = -0.10 to 0.14; *p* = 0.74), executive function (0.07, -0.07 to 0.20; *p* = 0.35), or processing speed (0.03, -0.08 to 0.14; *p* = 0.58). There were no associations between time spent in LPA on the previous day and cognitive scores in any cognitive domain (Table 2). For the majority of cognitive domains, there was no association between time spent in SB on the previous day and cognitive score. However, there was a minor reduction in working memory associated with increased time spent in SB. Each additional 30 min of SB on the previous day was associated with a reduction in working memory score of 0.05 SD (0.00 to 0.09; *p* = 0.03). Adjustment for sleep characteristics on the previous night did not substantively change point estimates for associations between the previous day’s MVPA and SB with cognitive performance (Table S3), though the association between MVPA and episodic memory was no longer statistically significant after adjustment.

**Table 2:** Associations of previous day physical activity and sedentary behaviour with cognitive performance (*N* = 76)

	**Physical activity and sedentary behaviour**					
	$$\beta$$ (95% CI)					
	**MVPA**	*p-value*	**LPA**	*p-value*	**SB**	*p-value*
Attention	0.02 (-0.10, 0.14)	0.74	-0.02 (-0.08, 0.04)	0.56	-0.01 (-0.06, 0.04)	0.70
Episodic memory	0.15 (0.01, 0.29)	0.03	-0.03 (-0.11, 0.05)	0.45	-0.04 (-0.10, 0.02)	0.17
Working memory	0.16 (0.03, 0.28)	0.01	0.02 (-0.05, 0.08)	0.61	-0.05 (-0.09, 0.00)	0.03
Psychomotor speed	0.09 (0.00, 0.18)	0.06	0.01 (-0.04, 0.05)	0.82	0.00 (-0.03, 0.03)	0.96
Executive function	0.07 (-0.07, 0.20)	0.35	0.01 (-0.05, 0.08)	0.72	-0.03 (-0.08, 0.02)	0.18
Processing Speed	0.03 (-0.08, 0.14)	0.58	0.00 (-0.05, 0.05)	0.87	0.01 (-0.03, 0.05)	0.66

Coefficients correspond to change in cognitive performance per 30-min increase in physical activity or sedentary behaviour on the previous day

Units are standard deviations. Adjusted for age, gender, education, mobility limitations, self-rated health, depressive symptoms, employment status, BMI, time-of-day of cognitive test taking, round of cognitive testing, weekend or weekday, habitual physical activity and sedentary behaviour, habitual sleep, previous cognitive score, sleep parameters at time $t-2$, and physical activity parameters at time $t$

Abbreviations: CI, confidence interval; MVPA, moderate-vigorous physical activity; LPA, light physical activity; SB, sedentary behaviour

### Sleep

After adjustment for physical activity and sedentary behaviour on the previous day, sleep duration on the previous night was associated with episodic memory and psychomotor speed (Table 3). Compared with participants with overnight sleep duration < 6 h on the previous night, participants with sleep duration ≥ 6 h had episodic memory scores 0.60 SD (0.16 to 1.03) higher (*p* = 0.008). Those with ≥ 6 h of sleep also had psychomotor speed 0.34 SD (0.04 to 0.65) faster than those with < 6 h of sleep (*p* = 0.03). Finally, there was a marginally significant association between overnight sleep duration and attention (*p* = 0.05). Compared with participants with < 6 h of sleep on the previous night, those with ≥ 6 h of sleep had attention scores 0.39 SD (0.00 to 0.79) higher.

**Table 3:** Associations between previous day sleep characteristics and cognitive performance (*N* = 76)

	**Sleep**					
	$$\beta$$ (95% CI)					
	**Sleep duration ≥ 6 hours**^**a**^	*p-value*	**REM sleep**^**b**^	*p-value*	**SWS**^**b**^	*p-value*
Attention	0.39 (0.00, 0.79)	0.05	0.13 (0.00, 0.25)	0.04	0.11 (-0.02, 0.23)	0.10
Episodic memory	0.60 (0.16, 1.03)	0.008	0.02 (-0.11, 0.16)	0.74	0.17 (0.05, 0.30)	0.008
Working memory	-0.16 (-0.59, 0.26)	0.45	-0.04 (-0.17, 0.09)	0.54	-0.03 (-0.18, 0.12)	0.67
Psychomotor speed	0.34 (0.04, 0.65)	0.03	0.05 (-0.04, 0.14)	0.26	-0.03 (-0.13, 0.06)	0.50
Executive function	0.23 (-0.21, 0.66)	0.31	0.03 (-0.10, 0.16)	0.67	0.03 (-0.12, 0.19)	0.67
Processing Speed	-0.13 (-0.46, 0.20)	0.45	0.05 (-0.05, 0.15)	0.31	0.01 (-0.09, 0.11)	0.88

*Abbreviations*: *CI* confidence interval, *REM* rapid eye movement, *SWS* slow wave sleep

^a^Reference: overnight sleep duration < 6 h

^b^Coefficient shown corresponds to change in cognitive performance per 30-min increase in sleep stage on the previous day

Units are standard deviations. Adjusted for age, gender, education, mobility limitations, self-rated health, depressive symptoms, employment status, BMI, time-of-day of cognitive test taking, round of cognitive testing, weekend or weekday, habitual physical activity and sedentary behaviour, habitual sleep, previous cognitive score, physical activity parameters at time $t-1$, and physical activity parameters at time $t$

There were also associations of time spent in REM sleep and SWS on the previous night with cognitive performance. Each additional 30 min of REM sleep on the previous night was associated with a 0.13 SD (0.00 to 0.25) increase in attention score (*p* = 0.04). Each 30-min increase in SWS was associated with a 0.17 SD (0.05 to 0.29) increase in score for episodic memory (*p* = 0.008). Each 30-min increase in SWS also corresponded to a 0.11 (-0.02 to 0.23) increase in attention score, but this increase did not reach statistical significance (*p* = 0.10). There was no other evidence of associations between sleep characteristics and cognitive scores for other cognitive domains (Table 3).

## Discussion

In this examination of associations of device-measured physical activity, sedentary behaviour, and sleep with next-day cognitive performance undertaken in 76 adults living in the UK aged 50-83 years, we found two key results. First, participating in more MVPA on the previous day was associated with better episodic and working memory performance, while more SB on the previous day was detrimental for working memory. These results were not substantively changed after taking into account sleep characteristics on the previous night. Second, independent of MVPA on the previous day, longer sleep duration overall on the previous night was associated with better performance for episodic memory and psychomotor speed, while more SWS was associated with better episodic memory and more REM sleep was associated with better attention scores. Taken together, the results suggest independent contributions of MVPA and sleep characteristics to next-day cognitive performance.

Several previous studies have examined associations between accelerometer-assessed physical activity and same-day cognitive performance. One study of 291 adults aged 40-70 years found that more physical activity than usual performed 20-60 min before cognitive testing was associated with better processing speed and better self-rated memory that evening, but was not associated with objectively-assessed visual memory [9], while another study of 90 adults aged 50 years and above found associations between physical activity and same-day executive function [11]. A study of 51 healthy participants aged 60 years and above failed to find an association between physical activity and same-day cognitive performance, but did find that physical activity accounted for up to 24% of intraindividual variability in cognitive performance during a five-day period depending on the cognitive task [12]. These findings of short-term associations between physical activity and cognitive performance are consistent with laboratory-based studies that suggest most neuropsychological changes induced by exercise last 6-90 min following a bout of exercise, while neurochemical stimulation occurs in the 20-120 min following exercise [5].

In the present study, we expand on these findings to show that MVPA was associated with improvements in episodic and working memory performance up to 24 h later, while SB was associated with reduced working memory function. Though the acute neurophysiological impacts of exercise may be short-term, other behavioural and neuropsychological benefits may linger, which could account for this result. Some evidence suggests enhanced positive mood states may be maintained for up to 24 h following exercise [42, 43], while fMRI measures implicated in memory retention remain elevated up to 48 h following MVPA [5, 44]. Our results are consistent with a previous study that examined associations between MVPA and next-day working memory performance in 64 preadolescents aged 10-13 years to show a between-person association of more MVPA with better working memory performance the following morning [10].

In the present study, we also examined the role of sleep. Sleep duration ≥ 6 h on the previous night was associated with improvements in episodic memory and psychomotor speed, and was marginally associated with better attention scores, which is in accordance with previous evidence suggesting sleep deprivation impacts hippocampal function [45–47], and reduces acuity and responsiveness to stimuli [32, 33, 48, 49]. The sleep-stage specific results underscore the importance of sufficient sleep duration for attention, where more REM was associated with higher attention scores and more SWS also corresponded to higher attention scores, though the results for SWS did not reach statistical significance. We also found that more SWS on the previous night was associated with better episodic memory performance, consistent with evidence suggesting SWS contributes to declarative memory function [17–20]. This may be one mechanism through which MVPA impacts memory on the following day, as exercise is associated with more time spent in SWS [22–25], supported by the slight attenuation of the association between MVPA and next-day episodic memory after adjustment for sleep characteristics that night; nonetheless, MVPA was associated with better next-day working memory performance even after adjusting for sleep, suggesting an independent contribution of MVPA to next-day memory function.

The main strength of our study is its novel micro-longitudinal study design, in which participants had daily cognitive tests during an accelerometer wear period, allowing us to expand on cross-sectional evidence examining associations between device-measured movement behaviours and cognitive performance. To our knowledge, our study is among the first to explicitly examine next-day cognitive performance in older adults using this study design. Though our sample size was small, each participant contributed multiple observations, increasing the power of our study. Our study was also able to take advantage of a novel polysomnography-validated sleep staging algorithm. While accelerometer-based sleep staging shows some discrepancies with polysomnography—in a validation study, the SleepNet algorithm used in the present study predicted REM sleep 17.1 min shorter and NREM sleep 31.1 min longer than polysomnography [30]—polysomnography is less feasible in free-living studies of movement behaviours. Finally, we reduced the likelihood of reverse causation or residual confounding driving our results as our study population was cognitively and clinically healthy and relatively homogenous in nature.

There are several limitations to this study. Our participants were highly active and cognitively healthy. It is possible we would have observed associations of movement behaviours with other cognitive domains with a more heterogenous sample or larger sample size, and further research is needed. Point estimates were nonetheless generally close to zero for cognitive domains that were not significantly associated with movement behaviours, suggesting lack of power did not lead us to miss associations in our analytic sample. An exception to this was for MVPA and executive function, where a modest benefit of MVPA did not reach significance; the suggestion that more MVPA is associated with improved next-day executive function is in line with previous results suggesting a same-day association between MVPA and executive function in older adults [11]. While the homogeneity of the analytic sample reduces some sources of bias, it also reduces the generalisability of the results; whether the findings will be similar in a study population experiencing neurocognitive disease or who are less active remains to be seen. Relying solely on device-based sedentary behaviour also meant that we were not able to examine types of sedentary behaviours, where cognitively-engaging sedentary behaviours have been associated with better cognitive performance, while other sedentary behaviours such as television watching are associated with worse cognitive performance [50]. Finally, we were not able to examine longer-term changes in cognitive function. The study should be reproduced in a larger and more diverse sample where longer periods of follow-up can be observed.

## Conclusions

With a rapidly ageing global population, identifying targets for intervention to promote healthy longevity is of increasing importance for public health. Maintenance of cognitive function is necessary to maintain active social participation and quality of life for older adults. While the acute cognitive benefits of physical activity are well-established, how long these benefits last and the role sleep plays is less clear. Though replication with larger sample sizes is needed, the present study puts forth evidence to suggest that the acute cognitive benefits of MVPA may persist for longer than previously thought, and good sleep quality may independently contribute to these benefits. The findings suggest that a lifestyle including MVPA alongside healthy sleep habits may contribute to supporting daily cognitive function for older adults.

## Supplementary Information

Supplementary Material 1.

## Data Availability

The dataset analysed during the current is are not publicly available due to institutional data sharing restrictions but summary data are available from the corresponding author on reasonable request.

## Abbreviations

REM: Rapid eye movement
SWS: Slow wave sleep
MVPA: Moderate-to-vigorous physical activity
LPA: Light physical activity
SB: Sedentary behaviour
SD: Standard deviation
BMI: Body mass index
CES-D: Center for Epidemiological Studies Depression
DAG: Directed acyclic graphs
CI: Confidence interval
