# Supplementary material for: Associations of accelerometer-measured physical activity, sedentary behaviour, and sleep with next-day cognitive performance in older adults: a micro-longitudinal study
Source: Int J Behav Nutr Phys Act. 2024 Dec 10;21:133. doi: 10.1186/s12966-024-01683-7 (PMC11629534; doi:10.1186/s12966-024-01683-7)
Supplement: Supplementary file 1 — Supplementary Material 1. [file 12966_2024_1683_MOESM1_ESM.docx]

**Associations of accelerometer-measured physical activity, sedentary behaviour, and sleep with next-day cognitive performance in older adults**

**Supplemental materials**

[Supplemental tables 2](#_Toc179370177)

[Table S1. Mobility limitations. 2](#_Toc179370178)

[Table S2. Coding of covariates in models. 3](#_Toc179370179)

[Table S3. Associations between previous day physical behaviours and cognitive performance after adjustment for previous night sleep (N=76). 4](#_Toc179370180)

[Supplemental figures 5](#_Toc179370181)

[Figure S1. Directed acyclic graph for physical activity and sedentary behaviour models. 5](#_Toc179370182)

[Figure S2. Directed acyclic graph for sleep models. 6](#_Toc179370183)

[Figure S3. Directed acyclic graph including all variables. 7](#_Toc179370184)

# Supplemental tables

## Table S1. Mobility limitations.

| **Participant reports difficulty with…** |
| --- |
| Walking 100 yards |
| Sitting 2 hours |
| Getting up from chair |
| Climbing 1 flight of stairs |
| Climbing several flights of stairs |
| Stooping/kneeling/crouching |
| Lifting/carrying 10 lbs |
| Picking up 5p coin |
| Reaching/extending arms above head |
| Pushing/pulling large object |

## Table S2. Coding of covariates in models.

| **Covariate** |  | **Coding type** |  | **Categories or range** |
| --- | --- | --- | --- | --- |
| Gender |  | Categorical |  | 1=Male  2=Female |
| Age (years) |  | Continuous |  | 50-83 |
| Highest educational qualification |  | Continuous |  | 1=No qualification  2=O-level  3=A-level  4=Further education below university degree  5=University degree  6=Postgraduate degree |
| Self-rated health |  | Continuous |  | 1=Poor  2=Fair  3=Very good  4=Excellent |
| Number of mobility limitations |  | Continuous |  | 0-10 |
| Employment status |  | Categorical |  | 0=Not employed  1=Employed |
| Body mass index (kg/m^2^) |  | Continuous |  | 16.9-38.8 |
| Depressive symptoms |  | Continuous |  | 0-20 |
| Time-of-day of test taking |  | Categorical |  | 1=Morning  2=Afternoon  3=Evening |
| Round of cognitive testing |  | Continuous |  | 1-3 |
| Weekend or weekday |  | Categorical |  | 1=Weekend  2=Weekday |

## Table S3. Associations between previous day physical behaviours and cognitive performance after adjustment for previous night sleep (N=76).

|  |  | **Physical behaviours**  $\beta$(95% CI) | | | | | | | |
| --- | --- | --- | --- | --- | --- | --- | --- | --- | --- |
|  |  | **MVPA** | *p-value* |  | **LPA** | *p-value* |  | **SB** | *p-value* |
| Attention |  | 0.03 (-0.10, 0.16) | 0.67 |  | -0.02 (-0.09, 0.04) | 0.50 |  | 0.00 (-0.05, 0.05) | 0.92 |
| Episodic memory |  | 0.12 (-0.04, 0.28) | 0.15 |  | -0.02 (-0.10, 0.05) | 0.58 |  | -0.03 (-0.08, 0.03) | 0.39 |
| Working memory |  | 0.17 (0.05, 0.29) | 0.008 |  | 0.03 (-0.04, 0.09) | 0.41 |  | -0.06 (-0.10, -0.01) | 0.02 |
| Psychomotor speed |  | 0.09 (0.00, 0.18) | 0.06 |  | 0.01 (0.04, 0.05) | 0.68 |  | 0.00 (-0.03, 0.03) | 0.99 |
| Executive function |  | 0.06 (-0.08, 0.20) | 0.38 |  | 0.02 (-0.05, 0.09) | 0.52 |  | -0.03 (-0.08, 0.02) | 0.20 |
| Processing Speed |  | 0.08 (-0.06, 0.22) | 0.26 |  | 0.02 (-0.05, 0.09) | 0.58 |  | -0.01 (-0.06, 0.04) | 0.63 |

Coefficients correspond to change in cognitive performance per 30-minute increase in physical behaviour on the previous day.
Units are standard deviations. Adjusted for age, gender, education, mobility limitations, self-rated health, depressive symptoms, employment status, BMI, time-of-day of cognitive test taking, round of cognitive testing, weekend or weekday, habitual physical activity, habitual sleep, previous cognitive score, sleep parameters at time $t-2$, physical activity parameters at time $t$, total sleep time, time spent in rapid eye movement, and time spent in slow wave sleep.
Abbreviations: CI, confidence interval; MVPA, moderate-vigorous physical activity; LPA, light physical activity; SB, sedentary behaviour.

# Supplemental figures

## Figure S1. Directed acyclic graph for physical activity and sedentary behaviour models.


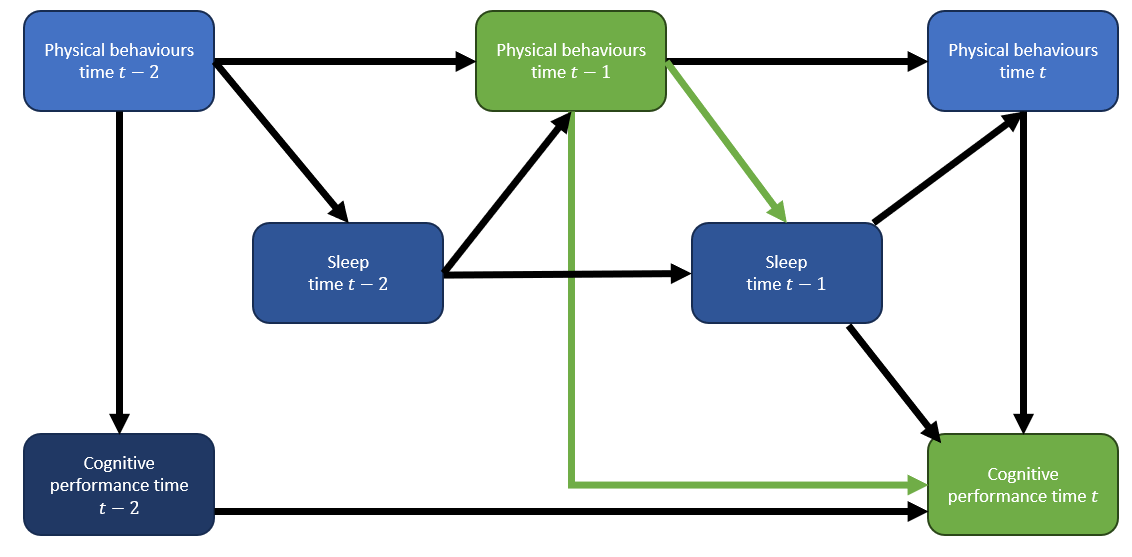


Green indicates pathway of interest.
Minimally sufficient adjustment set for total effect of physical behaviours (physical activity and sedentary behaviour) at $t-1$ on cognitive performance at $t$: cognitive performance at $t-2$ and sleep at $t-2$.
Models also adjusted for physical behaviours at $t$, habitual sleep and physical behaviours, and demographic, socioeconomic, health-related, and temporal covariates.

## Figure S2. Directed acyclic graph for sleep models.


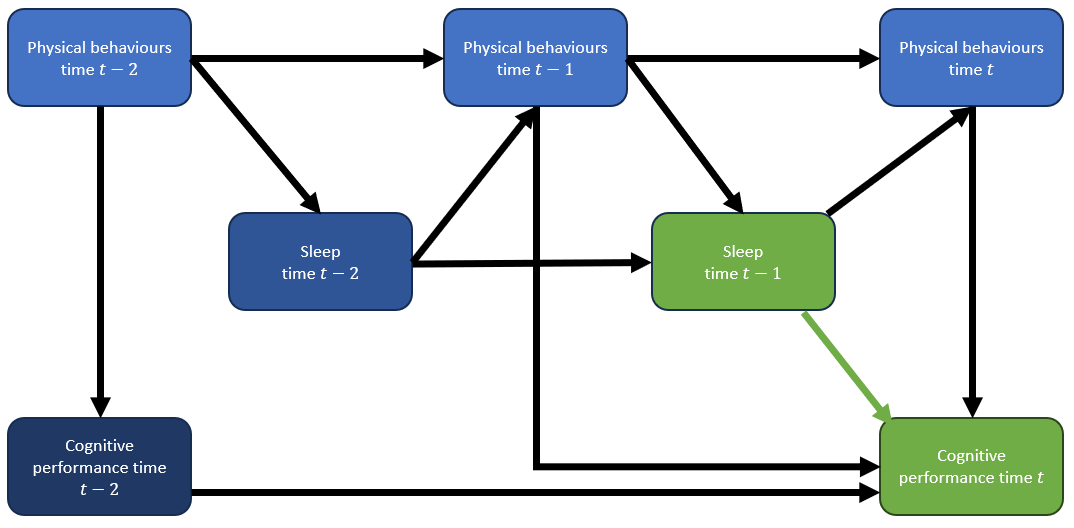


Green indicates pathway of interest.
Minimally sufficient adjustment set for total effect of sleep at $t-1$ on cognitive performance at $t$: cognitive performance at $t-2$ and physical behaviours (physical activity and sedentary behaviour) at $t-1$.
Models also adjusted for physical behaviours at $t$, habitual sleep and physical behaviours, and demographic, socioeconomic, health-related, and temporal covariates.

## Figure S3. Directed acyclic graph including all variables.


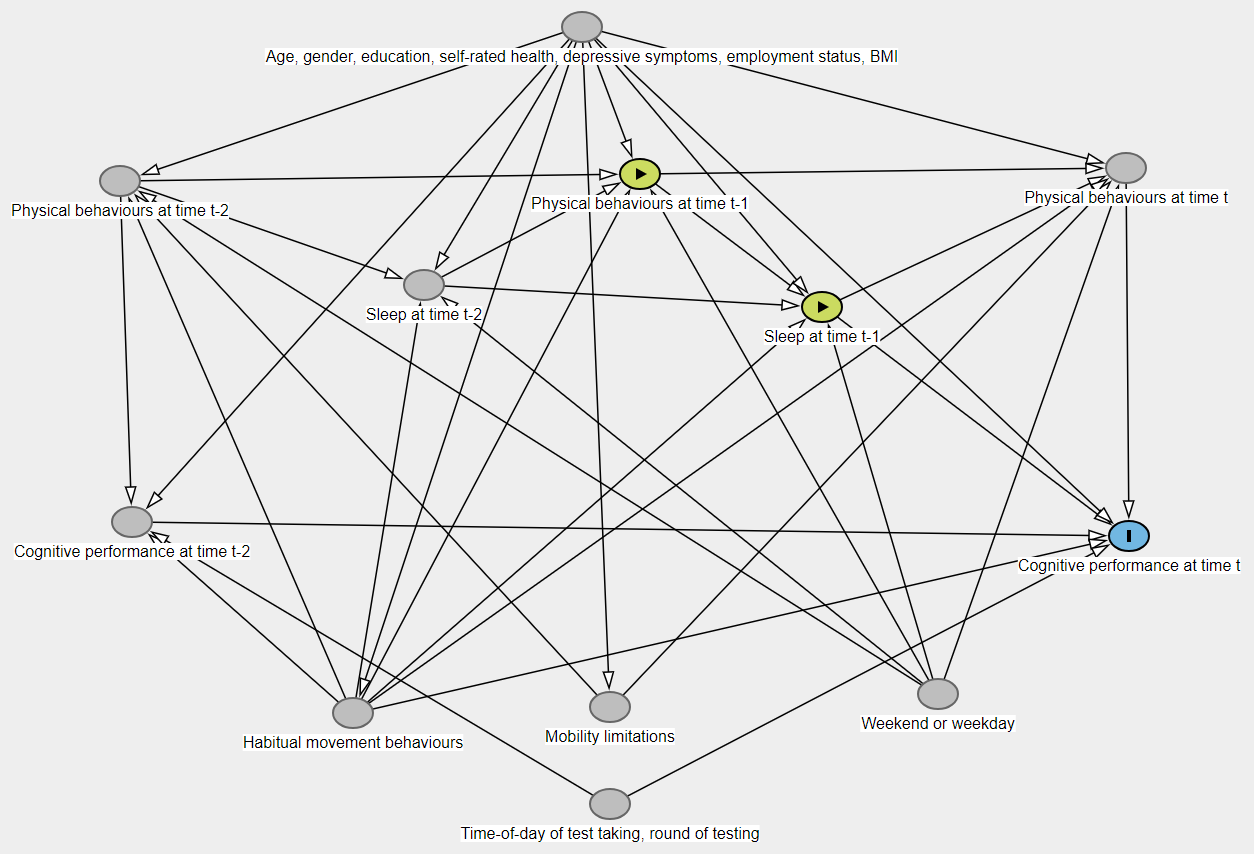


Green indicates exposure. Blue indicates outcome. Produced using *daggity* (<https://www.dagitty.net>).
